# Transcriptome analysis of the response of *Hypomyces chrysospermus* to cadmium stress

**DOI:** 10.3389/fmicb.2022.990693

**Published:** 2022-09-23

**Authors:** Yunan Wang, Chunze Mao, Yujia Shi, Xuejing Fan, Liping Sun, Yongliang Zhuang

**Affiliations:** Faculty of Food Science and Engineering, Kunming University of Science and Technology, Kunming, China

**Keywords:** *Hypomyces chrysospermus*, cadmium stress, transport systems, antioxidant enzymes, heavy metal tolerance, transcription factor

## Abstract

*Hypomyces chrysospermus* is a fungal parasite that grows on *Boletus* species. One isolated strain of *H. chrysospermus* from *B. griseus* was obtained and proved of strong ability to tolerate and absorb cadmium (Cd) by previous research. However, the molecular mechanisms of underlying the resistance of *H. chrysospermus* to Cd stress have not been investigated. This study aimed to assess the effect of Cd stress on the global transcriptional regulation of *H. chrysospermus*. A total of 1,839 differentially expressed genes (DEGs) were identified under 120 mg/l Cd stress. Gene ontology (GO) enrichment analysis revealed that large amounts of DEGs were associated with cell membrane components, oxidoreductase activity, and transport activity. KEGG enrichment analysis revealed that these DEGs were mainly involved in the translation, amino acid metabolism, transport and catabolism, carbohydrate metabolism, and folding/sorting and degradation pathways under Cd stress. Moreover, the expression of DEGs encoding transporter proteins, antioxidant enzymes, nonenzymatic antioxidant proteins, detoxification enzymes, and transcription factors was associated with the Cd stress response. These results provide insights into the molecular mechanisms underlying Cd tolerance in *H. chrysospermus* and serve as a valuable reference for further studies on the detoxification mechanisms of heavy metal-tolerant fungi. Our findings may also facilitate the development of new and improved fungal bioremediation strategies.

## Introduction

In the early 19th century, fungi were found to adsorb heavy metals. For instance, filamentous fungi, red mold, brewer’s yeast, and edible fungi were found to exhibit good heavy metal adsorption and tolerance (Isildak et al., 2007). The intensification of heavy metal pollution in the environment has resulted in a large number of fungi with high tolerance to heavy metal hazards. The high tolerance allows fungi to maintain their growth in the presence of heavy metals. Such heavy metal-tolerant fungi have become predominant in some areas with severe heavy metal pollution. At present, the main methods for controlling heavy metal pollution are chemical precipitation (Chen et al., 2018), ion exchange (Wong et al., 2014; Khanmohammadi et al., 2019), membrane separation, and adsorption (Adewale et al., 2018). Hence, much attention has been paid to highly effective and low-cost adsorption techniques using fungi for pollution remediation.

Cadmium (Cd) is one of the most concerning elements causing heavy metal pollution. Its main sources came are mining, electroplating, batteries, smelting and other industrial wastewater, waste gas, sludge, and agricultural fertilizers and pesticides, among others. Cd is ingested and absorbed by plants and animals and deposited in their bodies. Cd can enter the human body through the contaminated food chain and pose a threat to human health. Many studies have reported that several fungi, such as *Trichoderma asperellum*, *Funalia trogii*, *Penicillium* sp., and *Fusarium* sp., have a significantly higher ability to enrich Cd than to enrich other heavy metals (Zheng and Hou, 2019; Zhang et al., 2022). A previous study also revealed that Cd stress in *Lentinula edodes* could induce the selective expression of genes involved in transmembrane transport, glutathione (GSH) transport, and cytochrome P450 pathways and that these selectively expressed genes could increase the resistance of *L. edodes* to Cd stress (Yu H. et al., 2020). Cd resistance in *Morchella spongiola* may be related to catalytic activity, cell cycle control, and ribosomes assembly. Moreover, the expression of genes involved in major metabolic pathways, such as MAPK signaling, oxidative phosphorylation, pyruvate metabolism, and propionate metabolism pathways, in *M. spongiola* could be upregulated under Cd stress (Xu et al., 2021). Studies have revealed that *T. harzianum* could remove metals from the soil through bioremediation. In addition, in previous research, several upregulated spliceosome components of *T. harzianum* were found to cling to the fungus under Cd stress; the results unraveled the inhibition of carbohydrate-related proteins for the first time and demonstrated the susceptibility of the fungal parasite *T. harzianum* to Cd stress (Oshiquiri et al., 2020). Therefore, research on the identification of functional genes involved in Cd uptake and metabolism will be beneficial for application in genetic engineering to develop new technologies to treat Cd pollution.

*Hypomyces* is a genus of the most characteristic mycoparasites of diverse fungal hosts of agarics, boletes, russules, thelephores, and polypores in the family *Hypocreaceae* (Douhan and Rizzo, 2003). The parasites of *Hypomyces* usually cause systematic infection and mummification of the host fruiting bodies (Ouzhan et al., 2018). However, a symbiotic association of *H. chrysospermus* and *B. griseus* was found and identified by our previous study (Tian et al., 2022). We also confirmed that *B. griseus* had strong Cd-accumulated ability, even from natural habitats with low Cd contents (Bao et al., 2017; Sun et al., 2017; Xiao et al., 2021). A strain of *H. chrysospermus* was isolated from *B. griseus*. The isolated strain had a strong ability to tolerate Cd. The minimum inhibitory concentration of Cd of fungal growth was 200 mg/l. The Cd bioaccumulation capacity of the fungus reached 10.03 mg/g. The immobilization effects of the cell wall and acid compounds and antioxidant enzymes were employed by the fungus to alleviate the toxic effects of Cd. The fungus might be a potential bioremediation fungus for Cd contamination (Tian et al., 2022). However, the specific mechanism underlying Cd tolerance in *H. chrysospermus* remains unknown. In this study, the transcriptome of *H. chrysospermus* grown under 0 and 120 mg/l Cd stress was analyzed to identify differentially expressed genes (DEGs) associated with Cd adsorption and tolerance and to unravel the Cd stress response of *H. chrysospermus* in terms of the transcriptional expression of genes. Our findings would provide a theoretical basis for exploring the molecular mechanisms underlying Cd tolerance in *H. chrysospermus*.

## Materials and methods

### Mycelial culture and cd treatment

Fresh *B. griseus* was picked from the wild mushroom trading market in Shilin City, Yunnan Province, China, and an endophytic fungus was isolated on the same day. The isolated endophytic fungus was found to be *H. chrysospermus* after ITS sequence comparison. The culture was incubated in Potato Dextrose Water (PDB) containing 0 and 120 mg/l Cd at 28°C for 3 days with constant shaking at 120 rpm. Five replicate treatment groups were prepared for this experiment, and each group was analyzed. The mycelium was collected in a sterile environment, placed in lyophilization tubes (2 ml), rapidly frozen in liquid nitrogen, and stored at − 80°C for RNA extraction.

### RNA extraction, library preparation and sequencing

Total RNA was extracted from mycelial samples and was extracted from the tissue using TRIzol^®^ Reagent (Plant RNA Purification Reagent for plant tissue) according the manufacturer’s instructions (Invitrogen). Genomic DNA was removed using DNase I (TaKara). RNA degradation and contamination was monitored on 1% agarose gels. The integrity and purity of the total RNA quality was determined by 2,100 Bioanalyser (Agilent Technologies) and quantified using the ND-2000 (NanoDrop Technologies). Only high-quality RNA sample (OD260/280 = 1.8 ~ 2.2, OD260/230 ≥ 2.0, RIN ≥ 8.0, 28S:18S ≥ 1.0, >1 μg) was used to construct sequencing library.

RNA purification, reverse transcription, library construction and sequencing were performed at Shanghai Majorbio Bio-pharm Biotechnology Co., Ltd. (Shanghai, China) according to the manufacturer’s instructions (Illumina, San Diego, CA, United States). The transcriptome library was prepared following TruSeq^™^ RNA sample preparation Kit from Illumina (San Diego, CA, United States) using 1 μg of total RNA. Messenger RNA was isolated according to polyA selection method by oligo(dT) beads and then fragmented by fragmentation buffer firstly. Double-stranded cDNA was synthesized using a SuperScript double-stranded cDNA synthesis kit (Invitrogen, CA, United States) with random hexamer primers (Illumina). The synthesized cDNA was subjected to end-repair, phosphorylation and “A” base addition according to Illumina’s library construction protocol. Libraries were size selected for cDNA target fragments of 300 bp on 2% Low Range Ultra Agarose followed by PCR amplified using Phusion DNA polymerase (NEB) for 15 PCR cycles. After quantified by TBS380, paired-end RNA-seq sequencing library was sequenced with the Illumina NovaSeq 6,000 sequencer (2 × 150 bp read length).

### Quality control and data assembly

The raw sequencing data contained splice sequences, low-quality reads, sequences with high N contents (N represents uncertain base information), and sequences that were too short, which could seriously affect the quality of the subsequent correlation analysis. Therefore, SeqPrep^1^ and Sickle^2^ were used to check the quality of the raw sequencing data before analysis and to obtain high-quality clean data to ensure the accuracy of the subsequent analysis (Zhang et al., 2016). The accuracy of the results was ensured. For transcriptome analysis without reference genomes, after obtaining high-quality sequencing data through transcriptome sequencing, all sample clean data were assembled from scratch using Trinity and the assembly results were optimally evaluated (Grabherr et al., 2011).

### Analysis of DEGs and functional annotation

The experiment was performed with five biological replicates; therefore, DEG expression analysis was performed using DESeq2 software (*p*-adjust < 0.05, |log_2_FC| ≥ 1; Love et al., 2014). All transcripts obtained from this transcriptome sequencing were compared with those in six databases (NR, Swiss-Prot, Pfam, COG, GO, and KEGG databases) to obtain annotation information in each database and count the annotation status of each database. The software GOATOOLS^3^ was used to perform GO enrichment analysis of genes/transcripts in the gene set (Ye et al., 2018). GO standardizes the biological terminology of genes and gene products in different databases and provides a uniform qualification and description of gene and protein functions. KEGG is a knowledge base for the systematic analysis of gene function, linking genomic and functional data. Researchers can use the KEGG database to classify genes in a gene set according to the pathway they are involved in or the function they perform. Fisher’s exact test was performed, and the GO function and KEGG pathway were considered significantly enriched in the gene set when the corrected *p*-value (FDR) was less than 0.05.

### qRT-PCR validation

qRT-PCR was also performed on the same samples used for transcriptome analysis, primarily to verify the reliability of DEGs identified from the transcriptome sequencing data. In this experiment, four genes were selected for their potential involvement in improving Cd tolerance in *H. chrysospermus*; their expression was significantly upregulated (*p* < 0.05). Primer 5.0 was used to design primer sequences for the selected genes. The *β*-actin gene was selected as the internal reference gene (Suzuki et al., 2000; Negi et al., 2011). The primer sequences for the qRT-PCR experiments are listed in Table 1. qRT-PCR was performed using an ABI 7300 Fluorescent Quantitative PCR instrument (Applied Biosystems, United States). The reaction system for qRT-PCR consisted of the following: 0.8 μl of primer F, 0.8 μl of primer R, 6 μl of ddH_2_O, 2 μl of cDNA, 10 μl of 2 × ChamQ SYBR Color qPCR Master Mix, and 0.4 μl of 50 × ROX reference dye, making the final volume 20 μl. Each DEG was subjected to three technical replicates and three biological replicates in two treatment groups (0 and 120 mg/l Cd). The PCR amplification procedure was as follows: 5 min at 95°C and 40 cycles of 5 s at 95°C, 30 s at 55°C, and 40 s at 72°C. Ploidy was calculated using the 2^−ΔΔCT^ method.

**Table 1:** Primer sequences used in this study.

Gene id	Forward	Reverse
TRINITY_DN707_c0_g1	GCACCCGCTTCATCCTCA	CGGTTGGTCTCCCAGTCGTT
TRINITY_DN1814_c0_g1	TCATCTGTATCGCCGCATCT	CGGGTTCTCCTTGTCCGTAA
TRINITY_DN700_c0_g1	CAAACATCGCCCAACCTG	AACATGCGTGCAAACTCATAC
TRINITY_DN3517_c0_g1	TCATTGCCTGCGACACCC	AGCGACCCTTCTGACCACC
β-actin	CGACAATGGTTCCGGTATGTGCAA	ACGTAGGAGTCCTTCTGACCCATA

## Results and discussion

### Annotation of transcriptome sequencing data and assembly results

A total of 10,279 and 17,304 unigenes and transcripts were detected, respectively, with unigenes being the longest transcript and usually providing a better representation of the coding information of the genes. The clean data for each sample were above 6.45 Gb and the percentage of Q30 bases was above 93.85%, indicating that the sequencing data had sufficient quality to further justify the analysis. The GC content of the sequencing data was over 58%, also indicating the stability of the sequencing data (Supplementary Table S1). The correlation between the biological replicates of the samples in the experimental design was good, and the results verified the soundness of the experimental design (Supplementary Figure S1).

**Figure 1:**
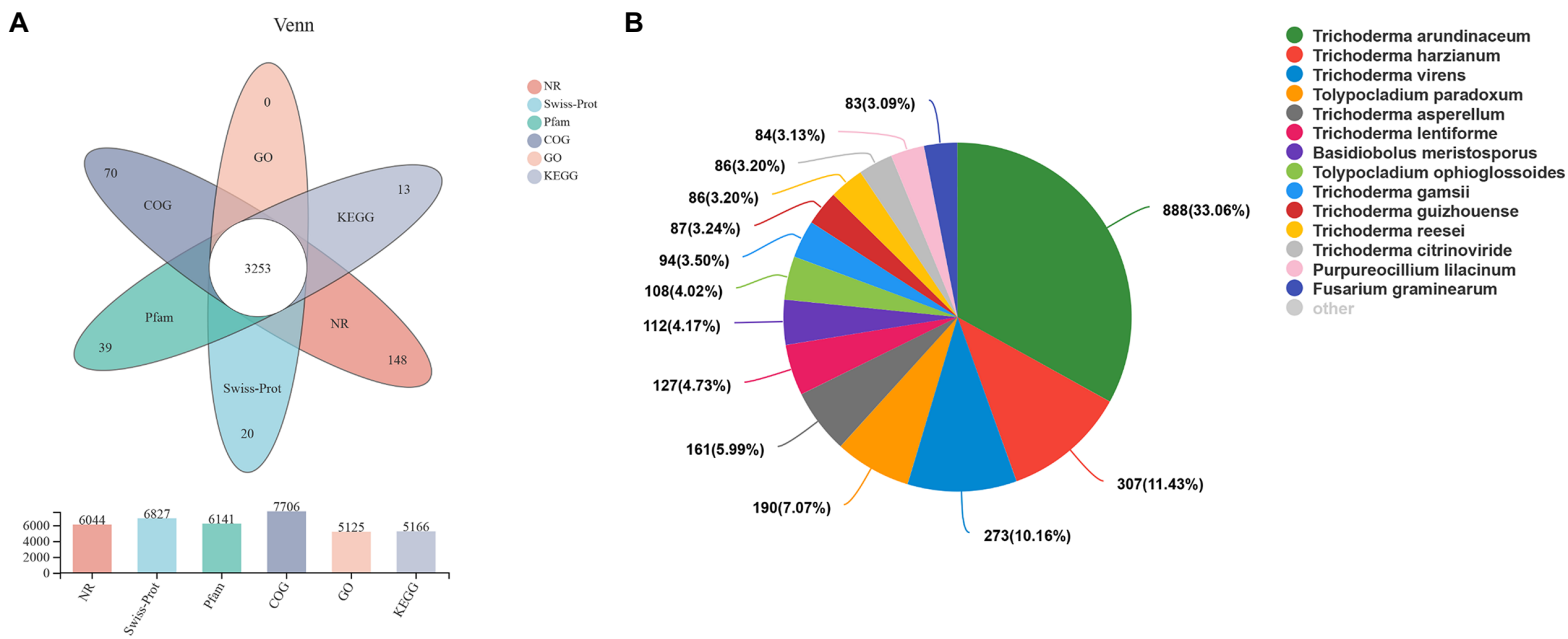
Transcriptome sequencing of *Hypomyces chrysospermus*. **(A)** Venn diagram of unigenes annotated in public databases, including Swiss-Prot, COG, KEGG, GO, and Pfam databases. **(B)** Species distribution of all annotated unigenes.

A total of 10,279 unigenes were obtained by non-reference splicing of the raw data and annotation results from the NR, Swiss-Prot, Pfam, COG, GO and KEGG databases (Figure 1A bar chart). A total of 3,543 genes were obtained in this study, of which a total of 3,253 (91.8%) genes were annotated in all six databases (Figure 1A Venn diagram). The similarity of the transcript sequences of *H. chrysospermus* to similar species was assessed by comparison with species in the NCBI_NR library. As shown in Figure 1B, the species closely related to *H. chrysospermus* could be annotated in the NR database. These mainly belonged to the genus *Trichoderma*, including *T. arundinaceum* (33.06%), *T. harzianum* (11.43%), and *T. virens* (10.16%).

### Functional enrichment analysis of DEGs

**Figure 2:**
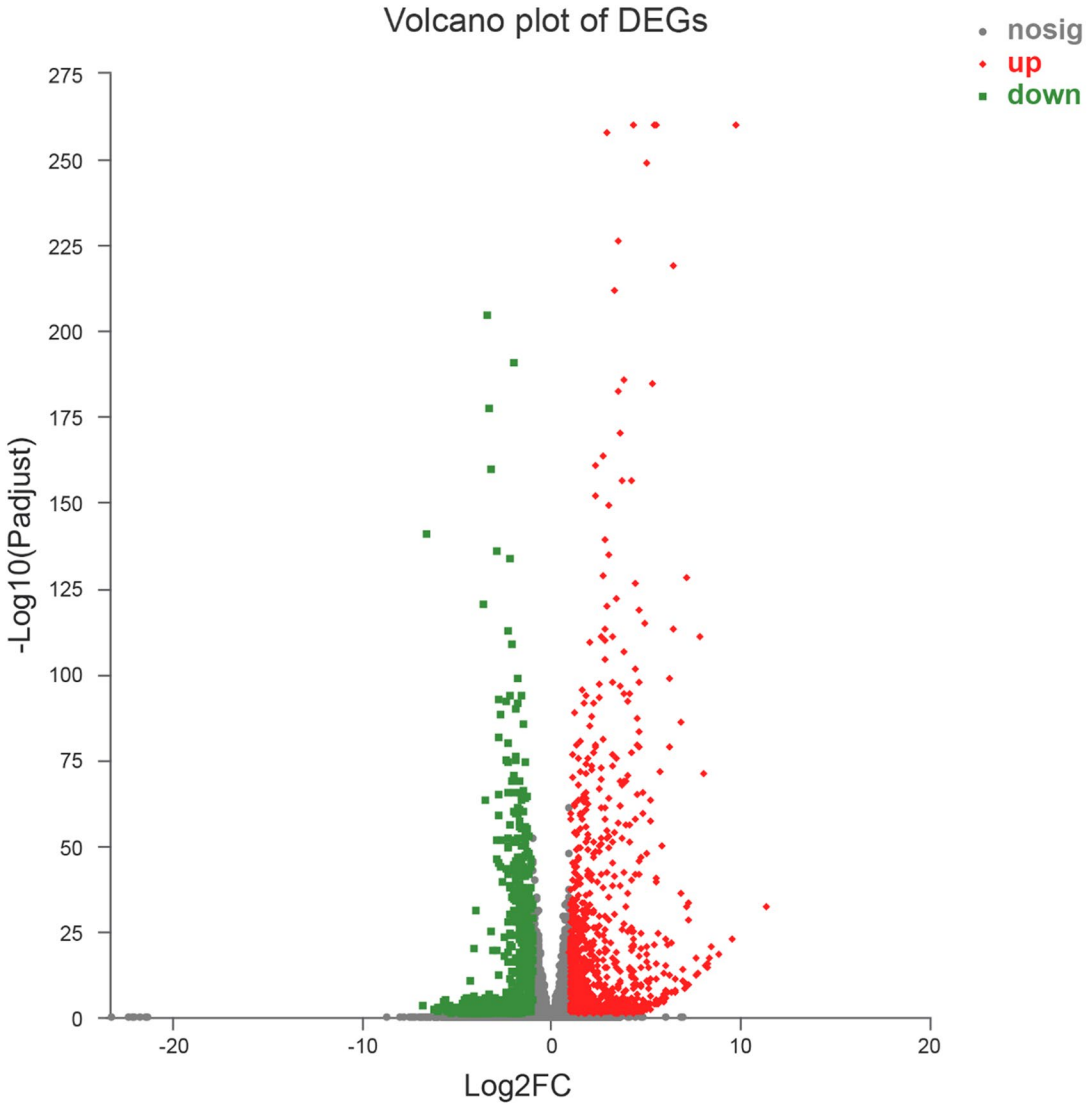
Volcano map of 1,839 differentially expressed genes.

**Figure 3:**
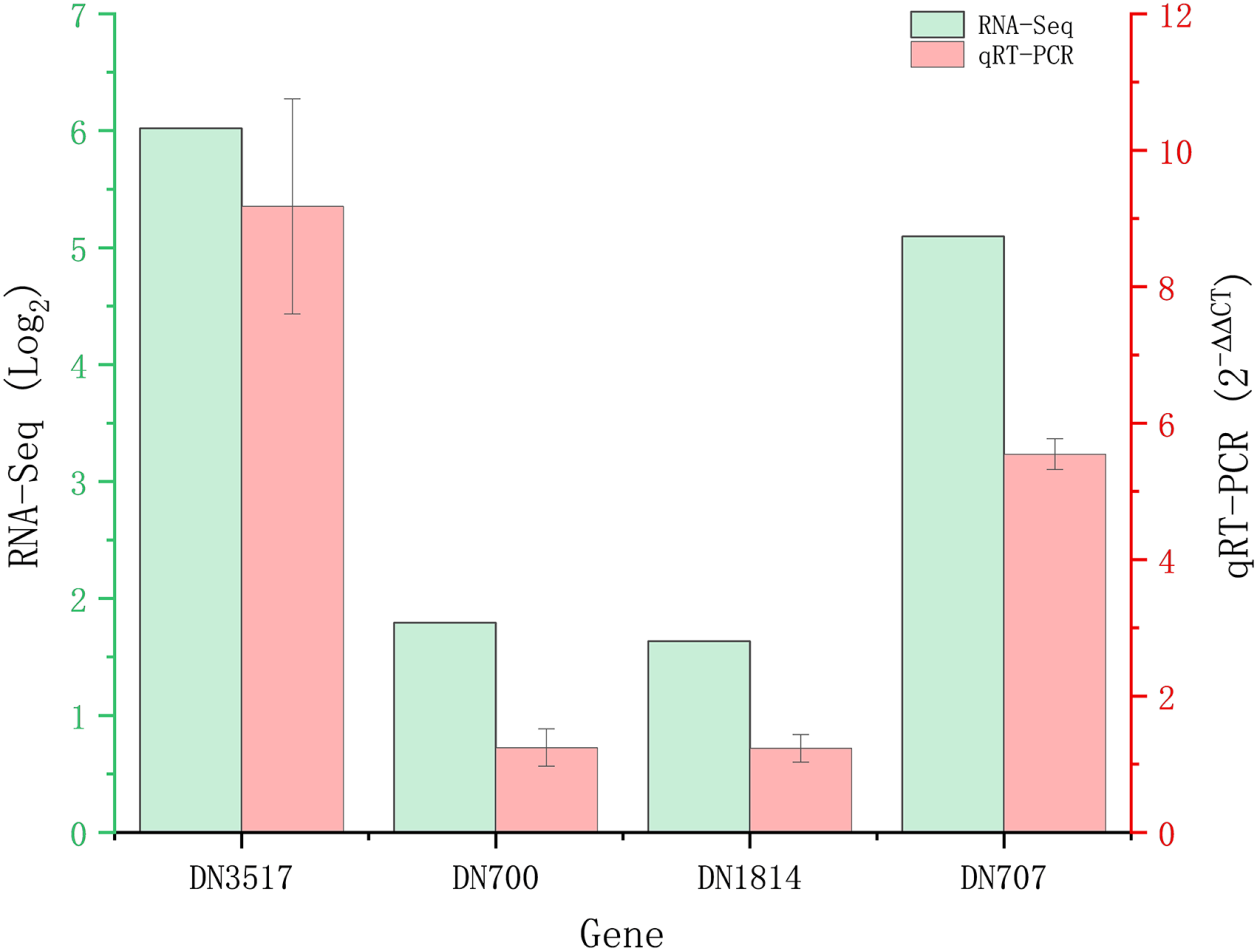
Validation of RNA-Seq results by quantitative real-time PCR (qRT-PCR). Four upregulated DEGs in *Hypomyces chrysospermus* were selected for validation.

A total of 1,839 DEGs were screened. Of these, 854 DEGs were downregulated, accounting for 46.44% of the total differential expression, and 985 DEGs were upregulated, accounting for 53.56% of the total differential expression (Figure 2). The expression levels of four genes related to Cd tolerance in *H. chrysospermus* were assessed by qRT-PCR in the two treatment groups to verify the accuracy of the transcriptome data obtained. The trends in the expression of the four DEGs obtained by qRT-PCR were similar to those in the transcriptome sequencing data (Figure 3; Supplementary Table S2). The Pearson correlation coefficient (*r*) was 0.974, indicating that the transcriptome sequencing data in the present study were reliable.

### Functional analyses of DEGs

**Figure 4:**
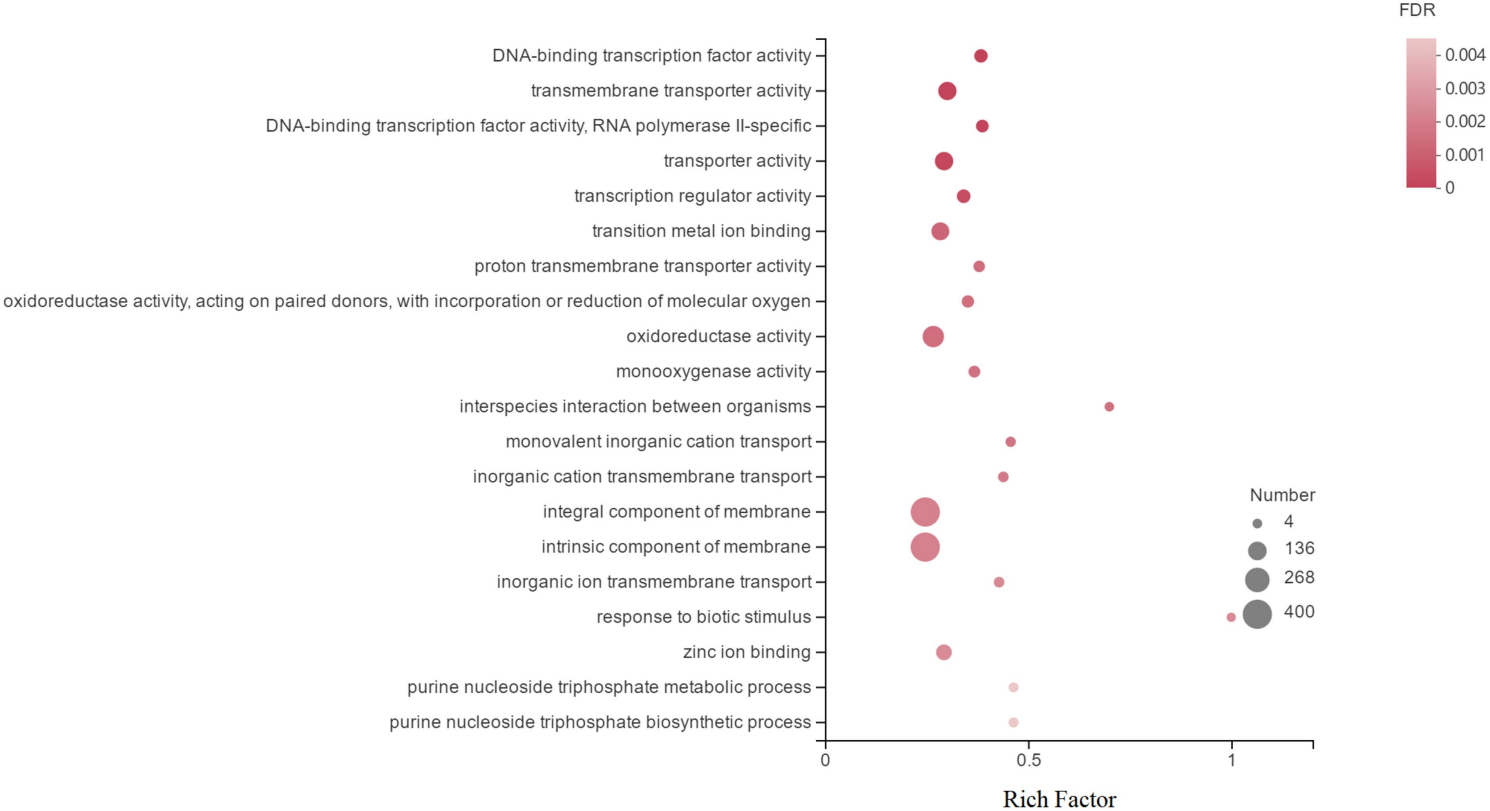
Top 20 enriched GO terms associated with DEGs in response to Cd stress. (The vertical axis represents the GO Term, the horizontal axis represents the Rich factor [the ratio of the number of samples enriched in the GO term to the background number; a larger Rich factor indicates greater enrichment], the size of the dots indicates the number of genes/transcripts in the GO Term, and the color of the dots corresponds to the different. The color of the dots corresponds to different FDR (*p* value corrected) ranges.)

GO is an international standardized gene function classification database that includes three relatively independent categories: cellular components, molecular functions, and biological processes. Figure 4 shows the top 20 DEGs in GO annotations. At the ontology level, DEGs induced by Cd in *H. chrysospermus* cells were mainly involved in cellular components and molecular functions. When these 1,839 DEGs were analyzed from the sublevel entries, the functional categories of the most GO-enriched genes were integral component of membrane, intrinsic component of membrane, oxidoreductase activity, transporter activity, transmembrane transporter activity and transition metal ion binding.

### Differentially expressed genes pathway analysis

To further identify the specific metabolic pathways that are altered in *H. chrysospermus* under Cd stress, a KEGG analysis of DEGs was performed under Cd stress. A total of 652 (35.5%) DEGs were annotated to different metabolic pathways. All Cd-induced DEGs could be classified into four categories of metabolic pathways. Of these, the metabolism category had the most enriched DEGs, more than 55% of the total annotated DEGs. The category with the least amount of enriched DEGs was environmental information processing, accounting for less than 3% of the total annotated DEGs. Table 2 shows the results of KEGG enrichment analysis of the top 20 enriched DEGs. At the metabolic pathways level, Cd-induced DEGs were mainly enriched in translation, amino acid metabolism, transport and catabolism, carbohydrate metabolism, and folding/sorting and degradation pathways. These pathways include oxidative phosphorylation, glycolysis/gluconeogenesis, amino sugar and nucleotide metabolism, glyoxylate and dicarboxylate metabolism, and purine metabolism.

**Table 2:** The 20 most significant pathways and numbers of DEGs.

Pathway id	Number	Description	First category	Second category
map03010	72	Ribosome	Genetic information processing	Translation
map00190	63	Oxidative phosphorylation	Metabolism	Energy metabolism
map03013	19	RNA transport	Genetic information processing	Translation
map04141	19	Protein processing in endoplasmic reticulum	Genetic information processing	Folding, sorting and degradation
map03040	17	Spliceosome	Genetic information processing	Transcription
map04144	17	Endocytosis	Cellular processes	Transport and catabolism
map00010	16	Glycolysis / gluconeogenesis	Metabolism	Carbohydrate metabolism
map04146	14	Peroxisome	Cellular processes	Transport and catabolism
map00020	13	Citrate cycle (TCA cycle)	Metabolism	Carbohydrate metabolism
map04011	13	MAPK signaling pathway: yeast	Environmental information processing	Signal transduction
map00520	13	Amino sugar and nucleotide sugar metabolism	Metabolism	Carbohydrate metabolism
map00380	12	Tryptophan metabolism	Metabolism	Amino acid metabolism
map03015	11	mRNA surveillance pathway	Genetic information processing	Translation
map00051	11	Fructose and mannose metabolism	Metabolism	Carbohydrate metabolism
map04113	11	Meiosis-yeast	Cellular processes	Cell growth and death
map00630	10	Glyoxylate and dicarboxylate metabolism	Metabolism	Carbohydrate metabolism
map04213	9	Longevity regulating pathway: multiple species	Organismal systems	Aging
map04933	9	AGE-RAGE signaling pathway in diabetic complications	Human diseases	Endocrine and metabolic disease
map00680	9	Methane metabolism	Metabolism	Energy metabolism
map03018	9	RNA degradation	Genetic information processing	Folding, sorting and degradation

### Expression of *Hypomyces chrysospermus* transport systems under cd stress

Metal transporter proteins are a class of transport proteins that are located on cell membranes and are involved in the uptake, transport, and compartmentalization of metal elements (Chen et al., 2017a). Extensive studies have identified a range of Cd- and its chelator-related transporter proteins, mainly the ATP-binding cassette (ABC) transporter protein, zinc/iron transporter protein (ZIP), and metal tolerance protein (MTP), using genetic engineering and modern molecular biology techniques (Zhang et al., 2018).

In this study, nine DEGs involved in the ABC transporter pathway were identified in *H. chrysospermus* under 120 mg/l Cd stress. The expression of these DEGs was significantly upregulated, and they mainly participated in the biosynthesis of ATM belonging to the ABC transporter protein B family and DPXA1/2 belonging to the ABC transporter protein D family (Supplementary Table S3). ABC transporter proteins are powerful transporters that could transfer inorganic ions, sugars, amino acids, lipids, lipopolysaccharides, peptides, and metal ions (Guo et al., 2022). Moreover, ABC transporter proteins play a crucial role in defense against virulence factors and drugs, particularly in sustaining dynamic metal ion homeostasis by transporting metal ions across the cell membrane in the form of ions/complexes (Higgins, 1992; Sanchez et al., 2001; Verrier et al., 2008; Do et al., 2018). For instance, DEGs regulating ABCC1 could reduce toxicity in *H. chrysospermus* by eliminating excess heavy metals from cells because the ABC transporter tolerance factors located in the mid-upper vesicle membrane can transport heavy metal toxins chelated with GSH into the vesicles for detoxification and can improve heavy metal tolerance in *H. chrysospermus* (Bruce, 2014). Hence, these upregulated DEGs may play an important role in conferring resistance to exogenous Cd (120 mg/l) in *H. chrysospermus*. These findings were similar to those of previous studies. Cai et al. assessed the ABC transporter protein gene OsABCG48 and found that the heterologous expression of OsABCG48 conferred Cd tolerance to corn wine fission yeast, *Arabidopsis* and rice (Cai et al., 2021).

The *ZIP* family is an important group of divalent metal transport proteins responsible for transporting metal ions, such as zinc, copper, manganese, iron, Cd, nickel, arsenic, and cobalt (Bashir et al., 2016). In this study, four DEGs associated with iron-regulated transporter (IRT) and zinc-regulated transporter (ZRT) proteins were significantly upregulated in *H. chrysospermus* in the presence of 120 mg/l Cd (Supplementary Table S3). Cd^2+^ has been reported to have the same outer electron configuration as Zn^2+^ and Fe^2+^. Moreover, the chemical properties of these ions are very similar. Hence, *H. chrysospermus* stimulated the expression of genes encoding IRT and ZRT and transported Cd^2+^ to maintain intracellular ion homeostasis (Tan et al., 2015; Zheng et al., 2022). These results were similar to those of a previous study, which reported that highly expressed DEGs involved in the ZIP–ZRT transporter pathways mediated the entry of higher levels of Cd^2+^ into the roots through the ZIP transporter in Cd-hyperaccumulating plants (Chen et al., 2017b; Zhu et al., 2018). Previous studies also revealed that the upregulated expression of IRT and ZRT increased Cd transport and accumulation in *Arabidopsis* under Cd stress (Becher et al., 2004).

In addition, DEGs associated with oligopeptide transporter (OPT), vesicular calcium transporter, MTP, and MFS multidrug resistance transporter regulatory pathways were upregulated in *H. chrysospermus* (Supplementary Table S3). The MFS family of transporter proteins is a superfamily of transmembrane transporters involved in the transport of drugs, metabolites, oligosaccharides, amino acids, and oxygen-containing anions, among others (Pao et al., 1998). Our results indicated that *H. chrysospermus* may have initiated its own detoxification mechanism to resist Cd stress and, to a certain extent, mitigate the harmful effects of the heavy metal Cd and improve its tolerance to Cd.

### Expression of antioxidant enzymes and antioxidant genes in *Hypomyces chrysospermus* under Cd stress

Enzymes play an important role in the abiotic stress response of microorganisms. Under normal conditions, the antioxidant enzymes superoxide dismutase (SOD), catalase (CAT), and peroxidase (POD) and the antioxidants GSH and ascorbic acid (AsA) in the cell scavenge the reactive oxygen species (ROS) produced during cellular metabolism, maintaining a very low level of ROS in the cell (Bansal et al., 2021). In this study, two CAT genes encoding glutathione S-transferase (GST) II (Log_2_FC = 3.374766182) and GSH synthetase (Log_2_FC = 1.087802885), which are involved in cysteine and methionine metabolism pathways, were significantly upregulated in response to 120 mg/l Cd treatment (Supplementary Table S4), possibly because excess Cd induced the production of large amounts of reactive oxygen radicals in the cells. To decrease oxidative stress and avoid oxidative damage to cell membrane lipids, these upregulated genes play an important role in the scavenging of ROS under Cd stress. Previous studies also revealed that the expression of POD, GSH synthetase, and photosynthesis-related proteins was significantly upregulated in *Piriformospora indica* under Cd treatment (Su et al., 2021). Moreover, the rhizobacteria *Serratia marcescens* S2I7 was found to have a GST-related mechanism to detoxify Cd (Kotoky et al., 2019).

### Expression of nonenzymatic antioxidant genes in *Hypomyces chrysospermus* under Cd stress

In fungi, heat shock proteins (HSPs) promote protein folding, stabilization, transport, and degradation and are therefore involved in the regulation of cell cycle processes and the activation of many key signaling enzymes (Roy and Tamuli, 2022). Thioredoxin (TRX) is a cofactor that acts as the electron donor for ribonucleotide reductase. It is involved not only in various physiological processes, such as the regulation of transcription factors, apoptosis, and antioxidant activity, but also in immune stress responses and redox reactions as a cofactor and an active growth factor. In this study, the expression of genes encoding HSP and TRX was found to be upregulated in *H. chrysospermus* under Cd stress (Supplementary Table S4), suggesting that the presence of heavy metals altered the redox state of the cells and induced the production of TRX and HSP. The small molecule proteins HSP and TRX could respond to the presence of high ROS in *H. chrysospermus* as nonenzymatic antioxidants. Hence, the upregulated expression of *hsp* and *trx* could increase the tolerance of *H. chrysospermus* cells to Cd by altering the redox status and chelating ions effectively. Many studies also found that exposure to Cd^2+^ could lead to ≥ 2–10-fold increase in HSP70 and HSP27 levels in organisms and that the increase in HSP70 responses induced by Cd damage may play a role in the protection of cell membranes (Yazdi et al., 2021). The increased levels of HSP70 in tobacco plants inoculated with *P. indica* suggested the involvement of HSP70 in increasing Cd tolerance in the plants (Hui et al., 2015). Moreover, whole-genome sequencing studies in *Rhizobium* JC1 revealed that heavy metal-responsive transcriptional regulators, TRX, and heavy metal transport/detoxification proteins play an important role in heavy metal adsorption and detoxification (Sun et al., 2021).

The expression of DEGs encoding GSH (Log_2_FC = 3.91050035; Supplementary Table S4) was found to be upregulated under 120 mg/l Cd stress; this may have improved the tolerance of *H. chrysospermus* cells to Cd stress. GSH is an important intracellular component responsible for heavy metal detoxification. It can react with heavy metals at the sulfhydryl group to form thiopeptide complexes and reduce the content of free-state heavy metals in cells (Orlando et al., 2021). Whole-genome resequencing and transcriptome analysis revealed that the expression of seven genes regulating GSH metabolism was altered in wild-type *Chlamydomonas reinhardtii* after exposure to Cd (Yu Z. et al., 2020).

### Expression of genes encoding metabolic detoxification enzymes in *Hypomyces chrysospermus* under Cd stress

Detoxification enzymes catalyze metabolic detoxification in cells in the presence of exogenous toxic substances. Of the several known detoxification enzymes, monooxygenases are the most important. Monooxygenases are multienzyme complexes having cytochrome P450 (CYP450) as their terminal oxidase that plays a key role in their catalytic function (Sligar, 2010). With an increase in Cd concentrations, nine DEGs encoding monooxygenases and seven DEGs encoding CYP450 were found to be significantly upregulated in this study. These results suggest that Cd stress can induce the production of CYP, which could help remove heavy metals and increase the tolerance of *H. chrysospermus* to Cd stress. Previous studies also reported CYP450 to be involved in Cd detoxification, salt tolerance, calcium signaling and homeostasis, and pathogen-triggered immunity in Chinese cabbage (Zhang et al., 2021). In addition, two molecular chaperones were found to be significantly upregulated in the *H. chrysospermus* transcriptome; these molecular chaperones may enhance the tolerance of cells to heavy metals by converting metal ion-induced misfolded and aggregated proteins into transiently active natural proteins (Qiu et al., 2006; Howell, 2013; Rauch et al., 2016).

### Expression of transcription factor genes in *Hypomyces chrysospermus* under Cd stress

Transcription factors are a group of proteins consisting of single or multiple structural domains that specifically bind to DNA. In this study, the expression of 14 DEGs encoding transcription factors, including one DEG encoding a fungus-specific transcription factor, one DEG encoding a heat shock factor, and four DEGs encoding C6 transcription factors, was upregulated in *H. chrysospermus* under Cd stress (Supplementary Table S5). Previous studies have reported that C6 transcription factors with zinc finger structures play an important role in response to heavy metal stress (Krishna et al., 2003; Jen and Wang, 2016) because the transcriptional co-activators with PDZ-binding motifs in zinc finger structures can confer resistance to various heavy metals by interacting with OsMYB34 and OsFHA9 transcription factors (Shalmani et al., 2021; Li et al., 2022). Hence, we hypothesized that the upregulated expression of DEGs encoding transcription factors could increase the tolerance of *H. chrysospermus* to Cd stress.

### Mechanisms underlying resistance of *Hypomyces chrysospermus* to Cd stress

Currently, the mechanisms underlying heavy metal tolerance in eukaryotes are divided into two main categories: intracellular tolerance mechanisms and extracellular tolerance mechanisms. Extracellular tolerance mechanisms mainly include the adsorption of heavy metals by the cell wall and the precipitation of heavy metals by extracellular secretion. Heavy metal adsorption by fungi is mainly attributed to cell wall components, such as chitin, dextran, cellulose, and proteins, and many functional groups that can bind to heavy metals, such as hydroxyl (–OH), carboxyl (–COOH), sulfhydryl (–SH), and amino (–NH_2_) groups. These groups can facilitate the adsorption of heavy metals on the cell wall and thus prevent heavy metal ions from entering the cell.

In this study, *H. chrysospermus* was found to grow under Cd stress, indicating that it can tolerate Cd toxicity. *H. chrysospermus* anchors Cd to the cell wall and then transports it into the cell through transport proteins or cytokinesis. Five DEGs encoding chitinase were upregulated in *H. chrysospermus*. The enriched Cd may have increased the expression of genes encoding chitinase, which may have helped transport Cd (Wang et al., 2021). In a study assessing Cd tolerance and accumulation in barley, transporter proteins and chitinases were also found to be involved in the transport of Cd (Cartagena Luna et al., 2020). In addition, fungi also secrete some organic acids that can bind to heavy metals and precipitate them, thereby preventing heavy metal ions from entering the cells (Salazar et al., 2020). Free amino acids have negatively charged hydroxyl and carboxyl functional groups that bind to heavy metal cations (Zhou, 1999). The amino acid metabolic pathways associated with Cd stress include arginine and proline metabolism; valine, leucine, and isoleucine biosynthesis; glycine and threonine metabolism; and GSH metabolism pathways (Pereira et al., 2002). In our study, three genes involved in arginine and proline metabolism, two genes involved in cysteine and methionine metabolism, and one gene catalyzing the linkage of glycine to tRNA were found to be significantly upregulated, suggesting that *H. chrysospermus* itself further resists exogenous Cd damage by increasing free amino acid metabolism under Cd stress. In addition, one gene encoding a capsular polysaccharide and two genes encoding iron carriers were upregulated in *H. chrysospermus* under Cd stress. Capsular polysaccharides can immobilize Cd by chelating and precipitating Cd^2+^ with carbonyl groups (–COO–), and siderophores can chelate heavy metal ions in the extracellular matrix. These properties help mitigate the toxic effects of Cd on the organism.

Heavy metal transport proteins also have a crucial effect on microbial metabolism under Cd stress. The main eukaryotic proteins tolerant to the heavy metal Cd are currently classified into three categories: transporter proteins, Cd-binding proteins, and proteins associated with Cd-binding proteins. In this study, nine genes involved in ABC transporter protein pathways were identified. ABC transporter proteins play an invaluable role in defense against virulence factors and drugs, particularly in sustaining dynamic metal ion homeostasis by transporting metal ions across the cell membrane in the form of ions/complexes. Therefore, the regulation of other genes associated with metal transporter proteins plays an important role in maintaining metal ion homeostasis in *H. chrysospermus* under Cd stress.

Under normal conditions, the antioxidant system, which consists of antioxidant enzymes and antioxidants, scavenges ROS produced during cellular metabolism. For example, the catabolism of CAT helps remove harmful substances, such as hydrogen peroxide, produced in the cell, which helps maintain a very low level of ROS in the cell (Xu et al., 2015). In this study, two CAT genes, one encoding GST II and the other encoding GSH synthetase (involved in cysteine and methionine metabolism), were upregulated under 120 mg/l Cd stress. These results indicate that the upregulated genes may induces the defense response of the antioxidant enzyme system in *H. chrysospermus* and mitigate the harmful effects of oxidative damage in cells.

In this study, one gene encoding HSP70 and two genes encoding TRX were upregulated in *H. chrysospermus* under Cd stress. These upregulated genes may have enhanced the tolerance of *H. chrysospermus* to Cd under severe Cd stress by altering the redox status of cells and chelating ions. In addition, the expression of genes encoding GSH was upregulated. The active group of GSH is a sulfhydryl group, which not only reacts with heavy metals to form thiopeptide complexes and to reduce the content of free heavy metals but also serves as an important intracellular component in the detoxification of heavy metals (Gadd, 1990). Therefore, increasing the level of GSH may help reduce the content of free heavy metals by forming complexes and improve the tolerance of organisms to heavy metals.

**Figure 5:**
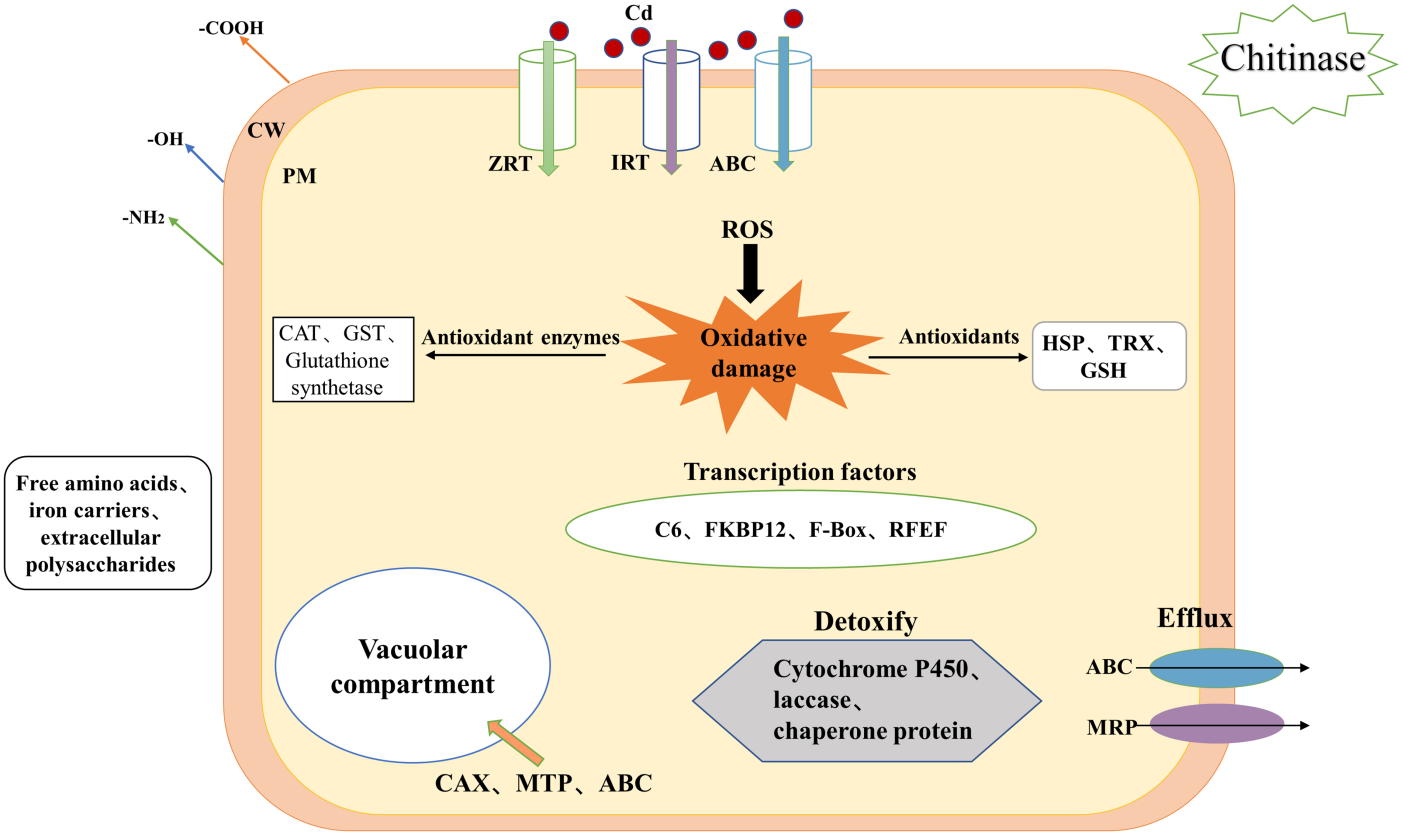
Mechanism underlying Cd accumulation and tolerance in *H. chrysospermus* cells. (CW: cell wall, PM: plasma membrane, Cd: cadmium, ROS: reactive oxygen species, ABC: ATP-binding cassette, ZRT: zinc-regulated transporter, IRT: iron-regulated transporter, MRP: multidrug resistance-associated protein, GSH: glutathione, HSP: heat shock protein, TRX: thioredoxin).

In summary, the regulatory network of *H. chrysospermus* in response to Cd stress is shown in Figure 5. The mycelium of *H. chrysospermus* increase its tolerance to Cd through multiple pathways. *H. chrysospermus* increase the level of chitinase to break down chitin in its environment and then uses it to synthesize its own cell wall. This improves the adsorption of Cd to the environment. Next, Cd mainly enters the cell through ABC, IRT, and ZRT metal transport proteins or other ion channels. The cell is under oxidative stress at this point. This leads to increased expression levels of genes encoding antioxidant enzymes (such as CAT, GST, and GSH synthetase) and nonenzymatic antioxidants (such as TRX and HSP). Moreover, genes involved in GSH metabolism are simultaneously upregulated, further enhancing the scavenging of ROS from the cells. In addition, genes related to free amino acid metabolism (e.g., proline and cysteine) are upregulated to bind to Cd and reduce its toxicity. Furthermore, the intracellular expression of the transcription factors C6 and FKBP12 is upregulated, potentially enhancing transcription factors that improve fungal tolerance when subjected to stress. Finally, *H. chrysospermus* upregulates the expression of the membrane-bound metal transport proteins OPT, MTP, and ABC, transferring Cd or Cd chelates into the vesicle or excreting the same from the cell. The combination of these mechanisms makes *H. chrysospermus* highly resistant to Cd stress.

## Conclusion

This study aimed to assess the transcriptome changes within the mycelium of *H. chrysospermus* under Cd stress. Our results provide a biomolecular explanation of how *H. chrysospermus* cells perceive and respond to Cd stress. Transcriptome sequencing revealed 1,839 DEGs in *H. chrysospermus* under 120 mg/l Cd stress. These DEGs mainly belonged to the integral component of membrane, intrinsic component of membrane, oxidoreductase activity, membrane activity, transporter activity, transmembrane transporter activity and transition metal ion binding functional categories. Based on these findings, we propose the regulatory network of *H. chrysospermus* in response to Cd stress. These findings can help comprehend the molecular mechanisms underlying Cd tolerance in *H. chrysospermus* in a more realistic and direct manner.

## Data availability statement

The datasets presented in this study can be found in online repositories. The names of the repository/repositories and accession number(s) can be found at: Sequence Read Archive (SRA) database (https://www.ncbi.nlm.nih.gov/sra/SRR20761392) accession number: PRJNA86458.

## Author contributions

YW conceived and supervised the study. CM designed the experiments. YS performed the experiments. XF analyzed the data. LS and YZ wrote the manuscript. All authors contributed to the article and approved the submitted version.

## Funding

The research was financially supported by the National Natural Science Foundation of China (31860421, 21767014) and Yunnan Major Scientific and Technological Projects (202202AG050009).

## Conflict of interest

The authors declare that the research was conducted in the absence of any commercial or financial relationships that could be construed as a potential conflict of interest.

## Publisher’s note

All claims expressed in this article are solely those of the authors and do not necessarily represent those of their affiliated organizations, or those of the publisher, the editors and the reviewers. Any product that may be evaluated in this article, or claim that may be made by its manufacturer, is not guaranteed or endorsed by the publisher.

## Footnotes

^1^ https://github.com/jstjohn/SeqPrep

^2^ https://github.com/najoshi/sickle

^3^ https://github.com/tanghaibao/GOatools

## Supplementary material

SUPPLEMENTARY FIGURE S1 Heat map of correlation between transcriptome samples of *Hypomyces chrysospermus*.
